# The negative impact on sleep for caregivers of children with intestinal failure on long-term parenteral nutrition

**DOI:** 10.1016/j.intf.2024.100021

**Published:** 2024-11-05

**Authors:** Christina Belza, Paul W. Wales, Darcy Fehlings, Wendy J. Ungar, Yaron Avitzur, Robyn Stremler

**Affiliations:** a Group for Improvement of Intestinal Function and Treatment (GIFT), Canada; b Institute for Health Policy Management and Evaluation, University of Toronto, Canada; c Child Health Evaluative Sciences, The Hospital for Sick Children Research Institute, Canada; d Division of General and Thoracic Surgery, Cincinnati Children’s Hospital Medical Center, Cincinnati, OH, USA; e Holland Bloorview Rehabilitation Hospital, Department of Pediatrics, Canada; f Division of Gastroenterology, Hepatology and Nutrition, The Hospital for Sick Children, Canada; g Lawrence S. Bloomberg Faculty of Nursing, University of Toronto, Canada

**Keywords:** Short bowel syndrome, Parenteral nutrition, Caregiver burden, Intestinal failure, Pediatrics, Informal caregiving

## Abstract

**Objectives:**

Survival rates for children with intestinal failure (IF) have improved with care provided in the home by family caregivers. Our objective was to compare total nocturnal sleep time (TST-N) in caregivers of children with IF on home PN to caregivers of children with a chronic medical condition.

**Methods:**

A comparative cross-sectional study of sleep was completed. All participants completed actigraphy and subjective measures of sleep (Pittsburgh Sleep Quality Index [PSQI], Multidimensional Assessment of Fatigue (MAF), Epworth Sleepiness Scale (ESS)). Univariate analysis was completed with an alpha value of < 0.05 considered significant.

**Results:**

Thirty-eight caregivers of children with IF and 29 caregivers of children without a chronic medical condition participated. Caregivers of children with IF achieved less sleep than caregivers of children without a chronic medical condition (7.0 vs 7.7 h, p < 0.01) resulting in 45 min less sleep per night. Caregivers of children with IF also demonstrated a shorter period of uninterrupted sleep (2.9 vs 4.2 h, p < 0.01), increased wake after sleep onset (49 vs 29 min, p < 0.01), increased night awakenings (8.9 vs 6.0, p < 0.01) and increased PSQI scores (7.7 vs 4.3, p < 0.01) compared to caregivers of healthy children.

**Conclusion:**

Caregivers of children with IF demonstrated decreased nighttime sleep amounts and sleep quality compared to caregivers of children without a chronic medical condition. This sleep debt may have significant consequences for the long-term health and wellbeing of caregivers and highlights the importance of further research to determine what supports are required to decrease the long-term impact for IF caregivers.

**Brief summary**.

**Current knowledge:** Children with intestinal failure on parenteral nutrition require significant medical support to ensure optimal health and due to their age, the majority of care is done in the home setting by family caregivers. There has been limited literature exploring the impact delivery of this care has on caregivers.

**Study impact:** This study demonstrates the significant interruption of sleep in caregivers who are providing care for children with intestinal failure dependent on parenteral nutrition support when compared to caregivers of children without chronic medical conditions. These findings open the discussion on how caregivers are impacted by providing care for children with complex medical needs and potential opportunities for supports or interventions to ensure caregivers needs are met.

## Introduction

Sleep is critical to optimal functioning and the amount of sleep an individual obtains significantly influences physical, emotional, and cognitive functioning [1], [2]. Current recommendations from the American Academy of Sleep Medicine and the Sleep Research Society advise that adults receive a minimum of 7 h of sleep per night [2]. Children with medical complexity (CMC) are a growing population. A recent study by the Canadian Institute of Health Information identified close to 100,000 CMC in Canada resulting in an incidence of 948 per 100,000 [3]. Caregivers of CMC are at an increased risk of sleep loss related to the increased demands of care, resulting in decreased sleep time and/or frequent interruptions through the night [4], [5]. Several published reports chronicling various chronic medical populations, have highlighted the challenges caregivers face in providing care and achieving adequate sleep [5], [6], [7], [8], [9]. Keilty et al. observed significantly decreased sleep duration in caregivers of children dependent on medical technology compared to caregivers of healthy children, in addition to higher rates of depressive symptoms and increased daytime sleepiness [5].

Care for children with pediatric intestinal failure (IF) has evolved significantly over the past two decades [10], [11]. The introduction of novel therapies has resulted in improved survival rates leading to an increased population of children requiring long-term parenteral nutrition (PN) support [10], [11], [12]. This unique group of patients has highly complicated medical needs requiring family caregivers to implement demanding care plans in the home setting with PN infusions, intravenous (IV) pumps, stoma care, IV medications and management of multiple medical devices. Family caregivers are the primary source of care in the home setting, providing an average of 29 hours of care per week on direct medical care [13]. The literature on the long-term impact related to stress, anxiety, depression and health-related quality of life (HRQoL) associated with providing care is limited but has highlighted the significant strain that caregivers of children with IF and their families experience [13], [14], [15]. Literature in adults on home PN report significant interruptions to sleep with the majority not meeting sleep recommendations with fewer hours of sleep per night, poorer sleep efficiency and longer wake after sleep onset [16]. Fatigue has also been reported in this patient population with the majority reporting severe or persistent fatigue [17].

To date, there have not been any evaluations of how sleep is impacted in caregivers providing informal care to children with IF. Our objective was to determine whether sleep quantity, quality, patterns, and daytime consequences of sleep loss vary significantly between caregivers of children with IF dependent on home PN compared to caregivers of healthy children.

## Methods

Participants were recruited between May 2019 and February 2020 and written informed consent was obtained from all participants and when indicated, assent was completed with the children with IF for the chart review component. Research ethics approval for the study was obtained from the Research Ethics Board at The Hospital for Sick Children (REB number: 1000062639).

### Study design

This was a cross-sectional comparative study with a primary objective to compare total nocturnal sleep time (TST-N) (in hours) in caregivers of children with IF on home PN (study group) to caregivers of children without a chronic medical condition (comparison group) over five nights. The secondary exploratory objectives compared the following outcomes between study and control groups: 1) longest uninterrupted sleep (in hours); 2) time awake after sleep onset (WASO) (in minutes); 3) number of nocturnal awakenings; 4) number of nights with less than 6 h of sleep; 5) self-reported sleep quality; 6) daytime sleepiness; 7) fatigue; and 8) exploratory models of factors related to sleep and patient management.

### Population and setting

The population of interest was caregivers of children with IF on long-term PN being followed by the intestinal rehabilitation program (IRP) at The Hospital for Sick Children in Toronto, Canada. The IRP at The Hospital for Sick Children follows children with IF from birth to 18 years of age, providing comprehensive care to patients in both inpatient and outpatient settings. Intestinal failure was defined as PN dependency for a minimum of 6 weeks with a diagnosis of short bowel syndrome (SBS), dysmotility or mucosal enteropathy or a residual small bowel length of less than 25 % expected for age at the time of the primary operation based on established norms for children with SBS [18]. Caregivers of children with IF on PN undergo a 2–3 week training program before discharge involving all aspects of central venous line management, preparation, and administration of PN and emergency procedures, in addition to other management tasks based on their child’s unique needs. Following hospital discharge, parents become the sole care providers for the child in the home and the majority do not have access to or receive limited nursing support from community providers [13].

Inclusion criteria for the study group included being the primary caregiver of a child aged 1–18 years with IF currently receiving home PN, primary residence in Ontario, Canada and proficiency to speak and read in English. Primary caregivers of both children with IF and caregivers of children without a chronic medical condition were defined as the individual in the home providing the majority of caregiving tasks for their child including childcare, meal preparation, and housework. Caregivers of children with IF were excluded if their children were within 6 weeks of discharge from their primary hospitalization or if they had a child in the home under 1 year of age. Recruitment was achieved through a mailed letter from the medical team, with further discussion and informed consent obtained during routine clinic appointments. The comparison group was recruited through posters in the hospital and a community-based pediatrician’s office. Inclusion criteria for the comparison group included being the caregiver of a child aged 1–18 years with no chronic health condition, primary residence in Ontario, Canada and proficiency to speak and read in English. Comparison group caregivers were excluded if there was a child in the home under the age of 1 year. The caregivers in the study group (caregivers of children with IF) were matched to a caregiver of child without a chronic medical condition based on the child’s age group: 1) toddler (ages 1–2 years); 2) preschool (ages 3–5 years); 3) school-aged (ages 6–12 years) and 4) adolescent (ages 13–18 years).

### Sample size

Sleep has not been evaluated in this patient population previously to inform what a clinically meaningful difference in TST-N would be. However, two previous studies of sleep in family caregivers of children on home ventilation used 60 min as a clinically important difference for sleep duration [5], [6]. The rationale for this difference was related to the potential to place the caregiver at risk for chronic sleep deprivation that is defined by the American Academy of Sleep Medicine as curtailed sleep that persists for a minimum of three months [5], [6], [19]. Meltzer & Mindell [8] in a study of caregivers of children on home ventilators, reported an average TST-N of 6.31 compared to 7.34 in caregivers of healthy children. Given this finding they felt that an additional hour of sleep per night would move family caregivers into the ideal 7–9 h of sleep per night resulting in reduction of sleep deprivation. Based on these previous studies, utilizing a two-sided t-test conducted in independent groups with an alpha value of < 0.05, a difference of 1 h in TST-N between group means, a standard deviation of 1.2 [6] and 90 % power required 32 participants per arm, while 80 % power would require 23 participants per arm. We chose to recruit based on a power of 90 % and allotted an additional 10 % for missing data or loss to follow-up that resulted in 36 participants per arm. The IRP at the The Hospital for Sick Children follows approximately 50 children on long-term home PN, with 40 meeting inclusion criteria for the study. Recruitment was suspended at the onset of the COVID-19 pandemic due to concerns that factors related to the pandemic would influence the results.

### Data collection

Data were collected from May 2019 to February 2020. Participants were given a package to be completed containing questionnaires (to be completed once by the participant), a daily sleep diary to be completed every day of the sleep collection period and an actigraphy device. Participants were instructed to wear the actigraphy device for a total of four days and five nights (daytime sleep data was not collected the first day of study). A minimum of three nights of completed actigraphy data was necessary for inclusion in the analysis. Once participants completed the questionnaires and actigraphy measurements the package was returned by mail or picked up by a member of the study team. Data from the questionnaires and sleep diaries were transcribed into an excel database by the primary investigator and double checked to ensure accuracy. Data was stored in a password protected file on a computer accessible only by members of the study team.

Caregivers in both the study group and comparison group were asked to complete assessments that included:

*Actigraphy* – Actigraphy involves wearing a watch-like device that measures activity and rest. Actigraphy was utilized for this evaluation to collect an objective measure of sleep patterns. Actigraphy is widely accepted for the evaluation of sleep patterns and sleep disorders by the American Academy of Sleep Medicine [20]. Multiple studies have demonstrated the validity of actigraphy compared to established methods for measuring sleep, including polysomnography, videosomnography and sleep diaries [16–23]. A minimum of 3 nights is recommended as the wear time by the American Academy of Sleep Medicine [20], [21]. In this study caregivers were asked to wear the actigraphy device on their non-dominant wrist. Actigraphy devices used were the Octagonal Basic Motionlogger (60 g, Ambulatory Monitoring, Ardsley, New York, USA) and were programmed to capture activity data at 1-minute epochs using the Cole-Kripke scoring algorithm (zero crossing mode) for analysis [22], [23].

*Sleep diary* – All caregivers completed a daily paper sleep diary. Caregivers recorded the time they went to sleep, wake times and other events that may have affected their ability to sleep. This aided in the interpretation of the actigraphy data. The sleep diary was adapted from the version utilized by Keilty, et al. [5] based on standardized tools used in previous investigations.

*Questionnaires* – Caregivers in both groups completed additional questionnaires at the end of their actigraphy sleep evaluation including: a demographic questionnaire, the Pittsburgh Sleep Quality Index (PSQI), the Epworth Sleepiness Scale (ESS) and the Multidimensional Assessment of Fatigue (MAF).

1. ***Demographic questionnaire*** – This questionnaire was developed by the primary investigator (CB) to collect information on gender, age, employment status, marital status, household income, and number of children and adults living in the home.
2. ***Pittsburgh Sleep Quality Index (PSQI)*** – This tool measured caregivers’ sleep quality and patterns. This tool is a validated 19-item subjective survey that measures seven different components related to sleep quality and patterns over the previous one-month. All 7 components are scored together to provide a total value with a score of 5 or more indicative of poor sleep quality [24], [25], [26], [27], [28], [29], [30], [31].
3. ***Epworth Sleepiness Scale (ESS)*** – This is a validated tool to assess daytime sleepiness in patients. It is a self-administered questionnaire with 8 questions to assess their chances of falling asleep while engaged in 8 common activities (i.e., sitting and reading, watching TV, passenger in a car for an hour). The normal range of scores is 0-10 with scores of 11-24 representing mild to severe levels of daytime sleepiness [26], [27], [32], [33], [34], [35], [36], [37].
4. ***Multidimensional Assessment of Fatigue (MAF)*** – The MAF is a 16-item scale measuring fatigue reported by participants over the previous 7 days. A higher total score indicates more severe fatigue, distress related to their fatigue or an impact on their daily activities. The use of the MAF has been reported in numerous studies showing good reliability and validity as reported in a recent systematic review [38].
5. ***Hospital Anxiety and Depression Score (HADS) and Parental Stress Index*** – Short form (PSI-SF). The PSI-SF is a 36-item tool evaluating parental stress. Questions are scored on a Likert scale with response options ranging from “Strongly Agree” to “Strongly Disagree” for most of the questions with three questions scored 1-5 with response options imbedded in the question [39]. The HADS is a 14-item scale to determine levels of anxiety (7 items) and depression (7 items) [40]. Questions are scored on a 4-point adjectival scale (0-3) with responses varying based on individual questions. The use of these tools were previously presented in [41], please refer to publication for tool descriptions and methods of collection.

*Child’s Clinical information* – Clinical information related to the child with IF (i.e., etiology, diagnosis, bowel anatomy and date of PN initiation) was collected through chart review by the primary investigator (CB).

### Statistical analysis

#### Demographics/primary objective

Demographic variables were evaluated through descriptive statistics with mean (sd) and frequency (proportions) for continuous and categorial variables, respectively. Comparison between groups was made using a student’s t-test for normally distributed data or Wilcoxon rank sum test for abnormally distributed data. Baseline differences between groups were controlled for as part of the secondary analysis using multivariable regression models. For the primary objective, sleep measurements were averaged over the number of nights completed. Summary statistics were completed to evaluate groups presented as means (SD) or medians (IQR) for continuous variables based on normality of data and frequency (proportions) for categorical variables. Normality of data was evaluated by review of histogram, QQ plots and the Shapiro-Wilk test of normality with a value of > 0.05 indicating normally distributed data. Multiple imputation was not used in the first analysis as there was minimal data missing. However, the analysis was complicated by the onset of the COVID-19 pandemic. Recruitment for the study was suspended because research activities were halted at our institution, as well as it was felt that the pandemic would impact sleep in caregivers of both groups and confound results. This resulted in unequal recruitment between groups. To account for the difference in group sizes for the actigraphy measurements of sleep, multiple imputation was completed to equalize group sizes and hypothesis testing performed using a paired t-test to evaluate differences between the data collected and the fully matched dataset. This analysis showed no significant differences from the presented study results; those data are not reported.

#### Secondary objectives

Our secondary analysis included data obtained from the actigraphy device including longest uninterrupted sleep time, WASO, number of nocturnal awakenings and sleep deprivation (number of nights with less than 6 h of sleep). Differences between groups were evaluated using a student’s t-test reporting means (sd) or Wilcoxon rank sum test with medians (IQR) based on normality of the data. Additionally, we evaluated the self-reported sleep quality obtained from the PSQI, daytime sleepiness reported on the Epworth Sleepiness scale and fatigue reported on the Multidimensional Assessment of Fatigue and compared between groups using a student’s t-test with means (sd) or Wilcoxon rank sum test with medians (IQR) for continuous data based on normality of data. Stress, anxiety and depression data were collected as part of a previous study with this sample [41] and included in this analysis. Finally, an exploratory multivariable analysis was conducted in caregivers of children with IF to evaluate factors that may impact their TST-N related to the child’s underlying medical condition using a backwards stepwise linear regression model. Variables included in the model were chosen a priori based on clinical expertise and included: age category (toddler, pre-school, school-age, or adolescent), number of clinic visits and hospitalizations in the previous year, number of consult services involved, IF type (SBS, mucosal enteropathy, dysmotility disorder), hours of PN infusion, total hours of informal care provided per week, presence of a stoma and administration of overnight gastrostomy feeds. A second linear regression model was completed to evaluate the impact of TST-N on mental health outcomes (stress, anxiety, and depression). Goodness of fit and model assumptions were evaluated, including R^2^ for model fit, normality of residuals, homoscedasticity, and multicollinearity (variance inflation factor and tolerance). A p-value of less than 0.05 for the initial univariate and final model was considered statistically significant and analyses were completed using SAS University Edition (Cary, NC).

## Results

### Demographics

**Fig. 1:**
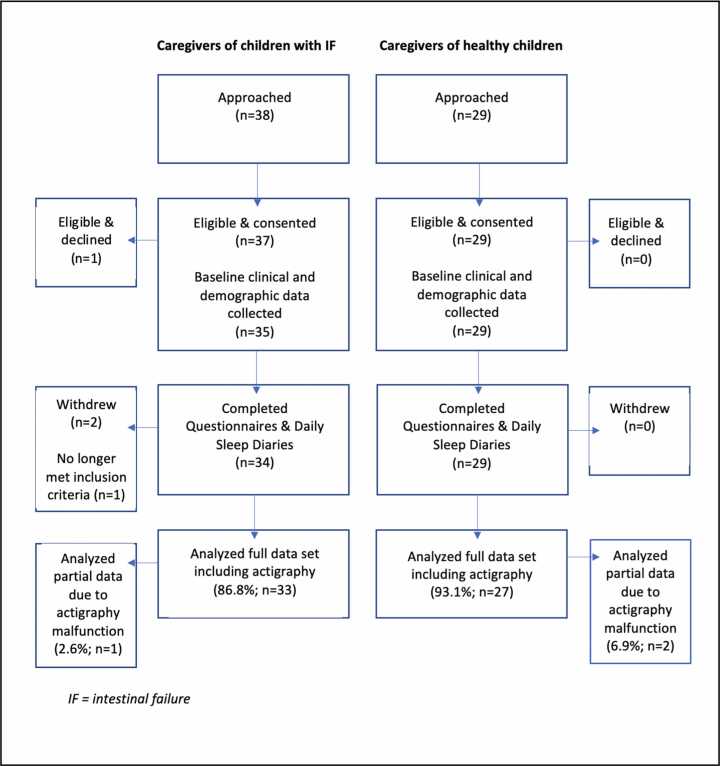
Schema of study recruitment.

Thirty-three caregivers of children with IF on home PN (study group) completed all data collection representing 87 % of those approached (N = 38) **(**Fig. 1**)**. One caregiver declined participation, 2 withdrew after consent (did not feel they had the time to complete required documentation) and 1 child came off PN before the family started data collection. One participant had incomplete documentation due to actigraphy malfunction. Twenty-nine caregivers of children without a chronic medical condition were approached and all consented to participate (100 %) **(**Fig. 1**)**. Two caregivers in the comparison group had incomplete documentation due to actigraphy malfunction and were not included in analysis. All participants included in the analysis had a minimum of 3 nights of complete actigraphy data available and no diary substitutions were made. Children in the study and comparison groups were comparable based on their age, sex, and age category (toddler, pre-school, school-age, and adolescent). Mothers were the predominant individual to complete the study accounting for 90.9 % of caregivers of children with IF and 92.6 % in the caregivers of children without a chronic medical condition group. Significant differences in employment status and income level existed between groups, with more IF caregivers earning below $60,000 CDN per year and more comparison group caregivers earning between $100,000–249,000 CDN per year. These results are highlighted in Table 1.

**Table 1:** Baseline demographics between groups of child and family caregivers. All values presented as frequency (%) unless otherwise indicated.

Variable	Caregivers of children with IF (n = 33)	Caregivers of healthy children (n = 27)
Child Information		
Age (years)	5.0 (2.0 −10.0)* *	6.0 (2.0 −10.0)* *
Age Category		
Toddler	11 (33.3)	9 (33.3)
Pre-School	6 (18.2)	4 (14.8)
School-Age	11 (33.3)	9 (33.3)
Adolescent	5 (15.2)	5 (18.5)
Sex		
Female	16 (48.5)	14 (51.9)
Male	17 (51.5)	13 (48.2)
Caregiver Information		
Age (years)	37.3 (8.2)*	39.1 (6.2)*
Sex		
Female	31 (93.9)	25 (92.6)
Male	2 (6.1)	1 (3.7)
Missing	0	1 (3.7)
Relationship to child		
Mother	30 (90.9)	25 (92.6)
Father	2 (6.1)	1 (3.7)
Foster/adoptive	1 (3.0)	0
Missing	0	1 (3.7)
Number of children < 18 living in home		
1	12 (36.4)	7 (25.9)
2	16 (48.5)	15 (55.6)
3	5 (15.2)	3 (11.1)
4	0	1 (3.7)
Missing	0	1 (3.7)
Number of adults > 18 living in home		
1	2 (6.1)	0
2	24 (72.7)	23 (85.2)
3 +	7 (21.2)	3 (11.1)
Missing	0	1 (3.7)
Diagnosed with a chronic health condition	5 (15.2)	3 (11.1)
Number of visits to health care provider for own needs in past 12 months	2.0 (1.0 −3.0)* *	1.5 (1.0 −6.0)* *
Marital status		
Married/living with partner	28 (84.8)	26 (96.2)
Single	3 (9.1)	0
Other	1 (3.0)	0
Missing	1 (3.0)	1 (3.7)
Highest level of schooling completed		
Elementary/grade school	0	1 (3.7)
High school	4 (12.1)	4 (14.8)
College/University	19 (57.5)	9 (33.3)
Graduate degree	9 (27.3)	11 (40.7)
Missing	1 (3.0)	2 (7.4)
Total family income (in Canadian dollars)		
< $25,000	4 (12.1)	0
$25,000 −39,000	4 (12.1)	0
$40,000 −59,000	2 (6.1)	0
$60,000 −99,000	4 (12.1)	3 (11.1)
$100,000 −249,000	10 (30.3)	16 (59.3)
> $250,000	7 (21.2)	7 (25.9)
Missing	2 (6.1)	1 (3.7)
Currently on maternity or parental leave	2 (6.1)	0
Missing	1 (3.0)	1 (3.7)
Current employment status of caregiver		
Working full time	11 (37.9)	18 (66.7)
Working part time	2 (6.9)	4 (14.8)
On critically ill childcare leave	1 (3.0)	0
Unemployed/looking for work	2 (6.1)	0
Disability/social assistance	2 (6.1)	0
Homemaker	7 (21.2)	3 (11.1)
Student	2 (6.1)	0
Not specified/prefer not to say	2 (6.1)	0
Other	0	1 (3.7)
Missing	4 (12.1)	1 (3.7)
Study nights completed		
3	1 (3.0)	0
4	4 (12.1)	4 (14.8)
5	28 (84.9)	23 (85.2)

IF=intestinal failure; IQR=interquartile range

*Mean (SD)

* *Median (IQR)

Children diagnosed with IF had varying etiologies with 75.8 % of the population having SBS, 15.2 % had mucosal enteropathies and 9.1 % had dysmotility disorders. Children had been on PN for just over 3 years (1239 day [IQR=432–3053]) with 65.6 % using two or more medical devices. Children with IF had a mean PN infusion time of 15.2 h (sd=3.7) with caregivers providing 34.3 (sd=17.8) hours of informal caregiving time per week. Only one caregiver reported receiving overnight nursing support during the sleep study collection and families had a median of 1.0 (IQR=0–10) hours of in-home nursing support per week. Table 2 highlights the clinical characteristics for the children with IF.

**Table 2:** Clinical characteristics of children with intestinal failure. All variables presented as frequencies (%) unless otherwise indicated.

**Variable**	Result (N = 32)
IF Category	
Short bowel syndrome	25 (75.8)
Mucosal enteropathy	5 (15.2)
Dysmotility	3 (9.1)
Gestational age (weeks)	35.5 (3.7)*
Birth weight (grams)	2522.1 (807.6)*
Small bowel (percentage) (n = 25)	13.0 (11.0 −23.0)* *
Large bowel (percentage) (n = 25)	50.0 (33.0 −75.0)* *
ICV Resected (n = 25)	10 (40.0)
Length of PN therapy (days)	1239 (432 −3053)* *
Additional diagnoses	16 (57.1)
Number of Consult services	2.0 (1.0 −2.0)* *
Number of Clinic visits (previous 12 months)	7.0 (5.0 −10.0)* *
Number of Hospitalizations (previous 12 months)	2.0 (1.0 −3.0)* *
Number of devices (CVC, gastrostomy, jejunostomy, stoma)	
1	11 (33.3)
2	13 (39.4)
3	8 (24.2)
Hours of PN infusion per day	15.2 (2.5)*
Hours of informal caregiver care per week	34.3 (17.8)*
Hours of nursing care per week	1.0 (0 −10.0)* *
Using in-home nursing services	9 (28.1)
Using overnight nursing support	1 (0.03)

CVC = central venous catheter; IF=intestinal failure; ICV=ileocecal valve; PN=parenteral nutrition; IQR=interquartile range

*Mean (SD)

* *Median (IQR)

### Sleep

Caregivers of children with IF had significantly fewer hours of TST-N than the comparison group (6.97±1.0 vs 7.72±0.8, p < 0.01) resulting in 45 min less sleep per night (Table 3). Compared to the comparison group, caregivers of IF patients demonstrated a significantly shorter period of uninterrupted nighttime sleep (2.9±1.1 h vs 4.2±1.5, p < 0.01), increased WASO (49.1±27.5 min vs 29.0±20.0, p < 0.01) and an increased number of awakenings overnight (8.9±4.8 vs 6.0±3.0, p < 0.01). Additionally, IF caregivers reported an increased number of sleep deprived nights (less than 6 h of sleep) compared to the comparison group (1.4±1.6 vs 0.3±0.7 nights, p < 0.01). Evaluation of subjective sleep measurement using the PSQI demonstrated significantly higher scores in caregivers of IF patients (7.7±3.0 vs 4.3±2.3, p < 0.01) with a higher proportion of study group participants demonstrating a score above the clinically important cut-off of 5 (84.4 % vs 30.8 %, p < 0.01). Results are highlighted in Table 3. Groups were also compared using multiple imputation to account for differences in group size with a paired analysis and demonstrated comparable results with no changes in significant findings (results not shown).

**Table 3:** Comparison of Outcomes Between Groups. All values presented as mean (SD) unless otherwise indicated.

**Variable**	Caregivers of children with IF (n = 33)	Caregivers of healthy children (n = 27)	p-value
**Sleep Measurements - Actigraphy**			
Total time in bed – night (hours)	7.7 (1.0)	8.2 (0.8)	0.06
Total sleep time – night (hours)	7.0 (1.0)	7.7 (0.82)	**< 0.01**
Longest uninterrupted sleep session (hours)	2.9 (1.1)	4.2 (1.5)	**< 0.01**
WASO (minutes)	49.1 (27.5)	29.0 (20.0)	**< 0.01**
Number of awakenings	8.9 (4.8)	6.0 (3.0)	**< 0.01**
Number of sleep deprived nights (less than 6 h of sleep)	1.4 (1.6)	0.3 (0.7)	**< 0.01**
Number of days with naps	0.5 (0.8)	1.0 (1.1)	**0.02**
Sleep efficiency percentage	89.5 (6.1)	94.3 (3.7)	**< 0.01**
**Sleep Measurements – Self-report**			
PSQI	7.7 (3.0)	4.3 (2.3)	**< 0.01**
PSQI			**< 0.01**
PSQI < 5 (good sleeper)	5 (15.6)^a^	18 (69.2)^a^	
PSQI > 5 (poor sleeper)	27 (84.4)^a^	8 (30.8)^a^	
ESS	7.5 (3.8)	6.4 (3.3)	0.25
MAF	29.6 (9.1)	18.6 (10.4)	**< 0.01**
Psychological Measurements -Self-report			
PSI-SF Total Stress	83.5 (27.4)	62.4 (12.9)	**< 0.01**
PSI-SF Total Stress Percentile	55.2 (29.2)	29.5 (19.6)	**< 0.01**
HADS Anxiety	9.4 (4.8)	6.5 (3.2)	**0.01**
HADS Depression	6.3 (4.3)	4.1 (2.6)	**0.03**

WASO=wake after sleep onset; PSQI=Pittsburgh Sleep Quality Index (abnormal score>5); ESS=Epworth sleepiness scale (abnormal score >10); MAF= Multidimensional Assessment of Fatigue; PSI-SF=Parental Stress Index-Short form (abnormal score >85th percentile); HADS=Hospital Anxiety and Depression Score (abnormal score >7).

a Frequency (%)

### Sleepiness, fatigue, depression and stress

Caregivers of children with IF reported higher levels of fatigue on the Multidimensional Assessment of Fatigue compared to comparison caregivers (29.6±9.1 vs 18.6±10.4, p < 0.01), but no significant difference in daytime sleepiness was observed using the Epworth Sleepiness Scale. Results are highlighted in Table 3.

### Multivariable model

A linear backwards stepwise regression model was completed to identify variables that may be significantly associated with hours of sleep (TST-N) among caregivers of children with IF. The only variable that remained significant in the model was the total hours of informal caregiving provided by caregivers (p < 0.01) (Table 4). A second linear regression model was completed to identify the impact of sleep on mental health outcomes (stress, anxiety, and depression). The model had a low R^2^ value (0.16) and was not significant (p = 0.39) despite total stress have a significant p-value (p = 0.05).

**Table 4:** Multivariable linear regression models.

Model 1: Variables that influence sleep in caregivers of children with IF^a^			
**Predictor**	**Adjusted beta coefficient (standard error)**	**Test statistic**	**p-value**
Omnibus F-test (F(ndf,ddf), pvalue)		5.21 (3,21)	**<0.01**
Total hours of informal caregiving per week	−2.35 (0.63)		**<0.01**
**Model 2: Impact of sleep on stress and anxiety**^b^			
Omnibus F-test (F(ndf,ddf), pvalue)		1.30 (4,37)	**0.39**
PSI-SF Total stress Score	1.05 (0.52)		**0.05**

a R^2^ =0.43

b R^2^ =0.16

## Discussion

Our results demonstrated significant differences in objective and subjective sleep measures between caregivers of children with IF on home PN compared to caregivers of children without a chronic medical condition placing caregivers at risk for negative outcomes related to inadequate and chronic sleep deprivation. While caregivers in both groups achieved similar amounts of time in bed, caregivers of children with IF obtained, on average, 45 min less sleep each night compared to caregivers of healthy children. This results in a significant sleep debt for caregivers of children with IF despite reaching the recommended 7 h of sleep per night. Caregivers of children with IF also demonstrated more WASO with an average of 49 min per night exceeding the insomnia criteria of 31 min [42]. Additionally, study caregivers demonstrated more nighttime awakenings, shorter uninterrupted stretches of sleep, poorer sleep quality and more sleep deprived nights. The daytime consequences of this sleep loss are demonstrated with higher levels of fatigue, effects on caregiver mood and elevated levels of anxiety.

### Sleep in caregivers of children with medical complexity

Previous studies evaluating objective sleep outcomes demonstrated similar results. Families caring for CMC experienced poorer sleep with caregivers of children dependent on medical technology demonstrating decreased TST-N, increased WASO and decreased sleep efficacy [5], [8]. Meltzer et al. evaluated caregivers of ventilator-assisted children compared to healthy controls and reported that the caregivers of children on home ventilators had later bedtime, shortened total sleep time, increased WASO and lower sleep efficacy compared to healthy controls [8]. They also demonstrated variability in wake time, increased WASO and lower sleep efficiency related to poorer HRQoL [8] similar to results highlighted by Keilty et al. [5]. Actigraphy has also been utilized in caregivers of children with autism compared to healthy controls with a significant decrease observed in total sleep time and variability of wake time, but not in other objective sleep measurements [7]. Our population obtained an average of 7 h of total sleep per night that is at the low end of the recommendations by the American Academy of Sleep Medicine and Sleep Research Society’s [2] recommendations for adults, but data collected on WASO and awakenings demonstrated that their sleep was fragmented with frequent interruptions and increased time awake after initially going to sleep. They also had a significantly higher proportion of nights with less than 6 h, demonstrating night-to-night variability in the amount of sleep they were able to obtain over the study period. This suggests that the medical needs of the child likely vary across nights, and caregiving may be shared by multiple caregivers (parents). Previously, parents reported frequent sleep interruptions related to medical care including pump malfunctions/occlusions, toileting, administering medications, enteral feeds and changing IV fluids [14].

Subjective measurements of sleep and other outcomes, including anxiety and depression, were evaluated in several quantitative and qualitative studies of caregivers with reports of daytime sleepiness and fatigue creating concerns regarding caregiver’s ability to function and the potential long-term effects on health, employment and ability to provide long-term care [4], [5], [6], [14], [43], [44]. There have also been previous reports highlighting the link between poor sleep patterns and increased rates of depression in caregivers of CMC [5], [9], [43]. Understanding directionality of the relationship between poor sleep and depression is challenging and research has suggested that both health problems need targeted intervention to prevent long-term consequences [45], [46]. While our population showed a significant difference in depression scores between groups the mean value did not reach a depression score of clinical significance, and it is important to note that these symptoms may have an influence on the evaluation of sleep quality. This would be important for future evaluation.

### Impact of in-home supports on sleep

This study highlighted the impact of providing complex care on caregivers’ sleep with only 9 families (28.1 %) accessing in-home nursing, one family utilizing overnight support (2.9 %) and a median of 1 h of nursing care per week. Families are often denied overnight supports as there are not active nursing interventions that are being completed and overnight interruptions for families are unpredictable (i.e. pumps beeping, diaper changes, etc). The minimal support that families were utilizing likely influenced caregivers’ sleep and overall functioning. A previous study in caregivers of children on home ventilators reported that those who received regular night nursing care had a significantly shorter sleep onset latency and while not statistically significant, those who received greater than 48 h of nursing care per week obtained an additional hour of sleep per night. They also found caregivers with fewer hours of nursing coverage overnight had depression scores above the clinical cut-off and increased daytime sleepiness [43]. In our previous work caregivers of children with IF on home PN highlighted significant challenges with accessing nursing resources in the community and inadequate training resulting in many families feeling that nighttime nursing support was not a helpful option for their family [14]. This gap in support results in caregivers taking on a large volume of care for prolonged periods of time with limited access to resources that would provide support and respite.

### Future directions

There have been significant advancements in IF outcomes which may be largely reflective of the IRPs ability to impart medical knowledge to caregivers and families ensuring they have the knowledge, skills and judgement to provide advanced medical care outside of the hospital. Caregivers of children with IF are an integral part of the IRP in ensuring successful intestinal rehabilitation that is often done at significant personal expense – physically, mentally and financially. As a health care system, we have provided children with IF access to exceptional medical care, resulting in significant improvements in outcomes for this patient population over the past two decades. IRPs can regularly evaluate the impact caregiving is having on families through discussion at outpatient visits to understand what challenges they are encountering in delivering care. Sleep measurement tools, such as smart watches, or validated tools for sleep and fatigue (PSQI, MSS, MAF) can also be utilized to provide an objective measurement or follow-up after changes in the medical plan. These discussions with family may identify opportunities for intervention/support (i.e., pump alarms, overnight bed changes). Caregivers have also demonstrated that they access minimal home supports to supplement the care they are providing. This deficiency places significant burden on families resulting in a multitude of effects on physical and mental health. Evaluation of the impact for families utilizing supports and services can identify future directions and opportunities for practitioners and families. Advocacy by practitioners and families aimed at policies and funding to improve the respite and support options for families would be an important next step to improve outcomes that require the high level of care provided by caregivers in this population. Additional avenues for research include determining the influence of a medicalized home on long-term health outcomes for caregivers and their children, focusing on sleep, stress, and overall health impacts. Evaluation of the relationship between anxiety and nighttime awakenings is another opportunity for future research targeting interventions, such as relaxation techniques that could facilitate quicker return to sleep and better sleep consolidation. It would also be important to evaluate caregiver sleep patterns in a larger multicenter cohort to determine factors that may influence sleep and other health outcomes to determine interventions that may have an impact in both children on and off PN. This may be particularly relevant in evaluating children who are actively undergoing intestinal rehabilitation compared to those that are chronically dependent on PN (i.e., dysmotility and CODE diagnoses).

### Strengths and limitations

Our evaluation of sleep was strengthened with high completion rates and minimal missing data for actigraphy, diary and questionnaires. However, this study represents the IF population at a single center in Toronto, Canada with practices and supports that may vary significantly from other programs and countries, reducing the generalizability of our results. Additionally, the cohort sizes were not equal and that could have influenced the study assumptions related to equal variances, statistical power, and type 1 error rates. This was evaluated by completing multiple imputation and re-evaluating results that demonstrated no changes in outcomes. An additional limitation relates to the collection of the minimum number of nights sleep using actigraphy. While this was an acceptable number of nights it may have been inadequate to evaluate fluctuations in sleep related to changes in the child’s care requirements or care provided by other care providers in the home. This study was cross-sectional, representing one particular period of time that prevents evaluation of predictive factors that may influence caregiver sleep over time. Our objective was to evaluate sleep in the primary caregiver, who in most of our participants was the child’s mother. Given that other studies have demonstrated a difference between mothers and fathers related to the amount of sleep they obtained [7], [47], it would be important to evaluate fathers’ sleep in future studies.

Selection bias may be present as we excluded caregivers with children under the age of 1 and those unable to speak/write English. We were able to mitigate this limitation with a high recruitment rate of those participants who met eligibility criteria. Questionnaires and diaries were clearly marked with instructions and time periods in question and participant packages were reviewed with caregivers at the time of enrollment.

## Conclusion

This study is an important step in demonstrating the impact that caring for children with complicated medical conditions in the home has on the caregivers and highlights the importance of further research to understand what supportive measures caregivers require to improve their sleep and decrease the impact on their health. It will also be important, in consultation with patients and families, to determine policy changes that need to be made at multiple levels of government to ensure adequate services are in place to support and sustain the caregivers providing long-term care.

## Ethical approval

Ethical approval was obtained at The Hospital for Sick Children (REB# 100006263). The data underlying this article cannot be shared publicly due to the privacy of individuals that participated in the study. The data will be shared on reasonable request to the corresponding author.

## Consent

Recruitment for caregivers of children with intestinal failure was achieved through a mailed letter from the medical team, with further discussion and informed consent obtained during routine clinic appointments. The comparison group was recruited through posters in the hospital and a community-based pediatrician’s office with informed consent being completed prior to study activities.

## Financial disclosure

All authors have no financial relationship (direct or indirect) relevant to this article to disclose.

## Clinical trial

This manuscript does not report on a clinical trial.

## Funding sources

Canadian Institute of Health Research (CIHR) Doctoral Award (FRN165748) Transplant and Regenerative Medicine Centre, The Hospital for Sick Children.

## CRediT authorship contribution statement

**Darcy Fehlings:** Writing – review & editing, Supervision, Methodology, Investigation, Conceptualization. **Wendy J Ungar:** Writing – review & editing, Supervision, Project administration, Methodology, Investigation, Conceptualization. **Yaron Avitzur:** Writing – review & editing, Supervision, Methodology, Investigation, Funding acquisition, Conceptualization. **Robyn Stremler:** Writing – review & editing, Supervision, Software, Resources, Project administration, Methodology, Formal analysis, Data curation, Conceptualization. **CHRISTINA BELZA:** Writing – review & editing, Writing – original draft, Project administration, Methodology, Investigation, Funding acquisition, Formal analysis, Data curation, Conceptualization. **Paul W Wales:** Writing – review & editing, Supervision, Resources, Project administration, Methodology, Data curation, Conceptualization.

## Declaration of Competing Interest

The authors declare the following financial interests/personal relationships which may be considered as potential competing interests: Christina Belza reports financial support was provided by Canadian Institutes of Health Research. If there are other authors, they declare that they have no known competing financial interests or personal relationships that could have appeared to influence the work reported in this paper.

## References

[1] Hirshkowitz M., Whiton K., Albert S.M. (Dec 2015). National Sleep Foundation's updated sleep duration recommendations: final report. Sleep Health.

[2] Consensus Conference P., Watson N.F., Badr M.S. (Aug 15 2015). Joint Consensus statement of the American academy of sleep medicine and sleep research society on the recommended amount of sleep for a healthy adult: methodology and discussion. J Clin Sleep Med.

[3] Information C.If.H. (2020).

[4] McCann D., Bull R., Winzenberg T. (Feb 2015). Sleep deprivation in parents caring for children with complex needs at home: a mixed methods systematic review. J Fam Nurs.

[5] Keilty K., Cohen E., Spalding K., Pullenayegum E., Stremler R. (Feb 2018). Sleep disturbance in family caregivers of children who depend on medical technology. Arch Dis Child.

[6] Meltzer L.J., Mindell J.A. (Sep 18 2006). Impact of a child's chronic illness on maternal sleep and daytime functioning. Arch Intern Med.

[7] Meltzer L.J. (May 2008). Brief report: sleep in parents of children with autism spectrum disorders. J Pedia Psychol.

[8] Meltzer L.J., Sanchez-Ortuno M.J., Edinger J.D., Avis K.T. (Mar 15 2015). Sleep patterns, sleep instability, and health related quality of life in parents of ventilator-assisted children. J Clin Sleep Med.

[9] Orta O.R., Barbosa C., Velez J.C. (2016). Associations of self-reported and objectively measured sleep disturbances with depression among primary caregivers of children with disabilities. Nat Sci Sleep.

[10] Fullerton B.S., Sparks E.A., Hall A.M., Duggan C., Jaksic T., Modi B.P. (Jan 2016). Enteral autonomy, cirrhosis, and long term transplant-free survival in pediatric intestinal failure patients. J Pediatr Surg.

[11] Duggan C.P., Jaksic T. (Aug 17 2017). Pediatric intestinal failure. N Engl J Med.

[12] Merritt R.J., Cohran V., Raphael B.P. (Nov 2017). Intestinal rehabilitation programs in the management of pediatric intestinal failure and short bowel syndrome. J Pediatr Gastroenterol Nutr.

[13] Belza C.U.W., Avitzur Y., Stremler R., Fehlings D., Wales P.W. Carrying the burden: Informal care requirements by caregivers of children with intestinal failure on home parenteral nutrition. *Ahead of publication*. 10.1016/j.jpeds.2022.05.049 35660493

[14] Belza C.P.C., Ghent E., Avitzur Y., Ungar W.J., Fehlings D., Stremler R., Wales P.W. “Line care governs our entire world”: Understanding the lived experience of caregivers of children with intestinal failure on long-term parenteral nutrition. *Ahead of publication*. 10.1002/jpen.2337 35088428

[15] van Oers H.A., Haverman L., Olieman J.F. (Aug 2019). Health-related quality of life, anxiety, depression and distress of mothers and fathers of children on Home parenteral nutrition. Clin Nutr (Edinb, Scotl).

[16] Dashti H.S., Godbole M., Chen A. (Sep 2022). Sleep patterns of patients receiving home parenteral nutrition: a home-based observational study. *JPEN*. J Parenter Enter Nutr.

[17] Huisman-de Waal G., Bazelmans E., van Achterberg T. (Sep 2011). Predicting fatigue in patients using home parenteral nutrition: a longitudinal study. Int J Behav Med.

[18] Struijs M.C., Diamond I.R., de Silva N., Wales P.W. (May 2009). Establishing norms for intestinal length in children. J Pediatr Surg.

[19] AAoS Medicine (2014). Third Edition ed. Int Classif Sleep Disord.

[20] Smith M.T., McCrae C.S., Cheung J. (Jul 15 2018). Use of actigraphy for the evaluation of sleep disorders and circadian rhythm sleep-wake disorders: an American academy of sleep medicine clinical practice guideline. J Clin Sleep Med.

[21] Martin J.L., Hakim A.D. (Jun 2011). Wrist actigraphy. Chest.

[22] Littner M., Kushida C.A., Anderson W.M. (May 1 2003). Practice parameters for the role of actigraphy in the study of sleep and circadian rhythms: an update for 2002. Sleep.

[23] Cole R.J., Kripke D.F., Gruen W., Mullaney D.J., Gillin J.C. (Oct 1992). Automatic sleep/wake identification from wrist activity. Sleep.

[24] Backhaus J., Junghanns K., Broocks A., Riemann D., Hohagen F. (Sep 2002). Test-retest reliability and validity of the Pittsburgh Sleep Quality Index in primary insomnia. J Psychosom Res.

[25] Buysse D.J., Reynolds C.F., 3rd, Monk T.H., Berman S.R., Kupfer D.J. (May 1989). The Pittsburgh sleep quality index: a new instrument for psychiatric practice and research. Psychiatry Res.

[26] Beaudreau S.A., Spira A.P., Stewart A. (Jan 2012). Validation of the Pittsburgh sleep quality index and the epworth sleepiness scale in older black and white women. Sleep Med.

[27] Spira A.P., Beaudreau S.A., Stone K.L. (Apr 2012). Reliability and validity of the Pittsburgh Sleep Quality Index and the Epworth Sleepiness Scale in older men. J Gerontol A Biol Sci Med Sci.

[28] Grandner M.A., Kripke D.F., Yoon I.Y., Youngstedt S.D. (Jun 2006). Criterion validity of the Pittsburgh Sleep Quality Index: investigation in a non-clinical sample. Sleep Biol Rhythms.

[29] Mollayeva T., Thurairajah P., Burton K., Mollayeva S., Shapiro C.M., Colantonio A. (Feb 2016). The Pittsburgh sleep quality index as a screening tool for sleep dysfunction in clinical and non-clinical samples: a systematic review and meta-analysis. Sleep Med Rev.

[30] Nicassio P.M., Ormseth S.R., Custodio M.K., Olmstead R., Weisman M.H., Irwin M.R. (2014). Confirmatory factor analysis of the Pittsburgh Sleep Quality Index in rheumatoid arthritis patients. Behav Sleep Med.

[31] Bush A.L., Armento M.E., Weiss B.J. (Aug 30 2012). The Pittsburgh Sleep Quality Index in older primary care patients with generalized anxiety disorder: psychometrics and outcomes following cognitive behavioral therapy. Psychiatry Res.

[32] Heaton K., Anderson D. (2007). A psychometric analysis of the Epworth Sleepiness Scale. J Nurs Meas.

[33] Janssen K.C., Phillipson S., O'Connor J., Johns M.W. (May 2017). Validation of the Epworth Sleepiness scale for children and adolescents using rasch analysis. Sleep Med.

[34] Johns M., Hocking B. (Oct 1997). Daytime sleepiness and sleep habits of Australian workers. Sleep.

[35] Johns M.W. (Dec 1994). Sleepiness in different situations measured by the Epworth Sleepiness Scale. Sleep.

[36] Johns M.W. (Aug 1992). Reliability and factor analysis of the Epworth Sleepiness Scale. Sleep.

[37] Johns M.W. (Dec 1991). A new method for measuring daytime sleepiness: the Epworth sleepiness scale. Sleep.

[38] Belza B., Miyawaki C.E., Liu M. (Apr 1 2018). A systematic review of studies using the multidimensional assessment of fatigue scale. J Nurs Meas.

[39] Abidin R.R. (2012). Parenting Stress Index.

[40] Zigmond A.S., Snaith R.P. (Jun 1983). The hospital anxiety and depression scale. Acta Psychiatr Scand.

[41] Belza C.A.Y., Ungar W.J., Stremler R., Fehlings D., Wales P.W. Stress, anxiety and health-related quality of life in caregivers of children with intestinal failure on parenteral nutrition. *Ahead of publication*. 10.1002/jpen.2461 36336350

[42] Lichstein K.L., Durrence H.H., Taylor D.J., Bush A.J., Riedel B.W. (Apr 2003). Quantitative criteria for insomnia. Behav Res Ther.

[43] Meltzer L.J., Boroughs D.S., Downes J.J. (Aug 2010). The relationship between home nursing coverage, sleep, and daytime functioning in parents of ventilator-assisted children. J Pedia Nurs.

[44] Steele R., Davies B. (Dec 2006). Impact on parents when a child has a progressive, life-threatening illness. Int J Palliat Nurs.

[45] Fang H., Tu S., Sheng J., Shao A. (Apr 2019). Depression in sleep disturbance: a review on a bidirectional relationship, mechanisms and treatment. J Cell Mol Med.

[46] Franzen P.L., Buysse D.J. (2008). Sleep disturbances and depression: risk relationships for subsequent depression and therapeutic implications. Dialog- Clin Neurosci.

[47] Meltzer L.J., Flewelling K.D., Jump S., Gyorkos E., White M., Hauk P.J. (Apr 2020). Impact of atopic dermatitis treatment on child and parent sleep, daytime functioning, and quality of life. Ann Allergy Asthma Immunol.

